# Decoding spiking activity in V4, but not V1, correlates with behavioural performance in perceptual learning task

**DOI:** 10.1186/1471-2202-14-S1-P385

**Published:** 2013-07-08

**Authors:** Scott C Lowe, Xing Chen, Mark CW van Rossum, Stefano Panzeri, Alexander Thiele

**Affiliations:** 1 Institute for Adaptive and Neural Computation, School of Informatics, University of Edinburgh, Edinburgh, EH8 9AB, UK; 2 Institute of Neuroscience, Newcastle University, Newcastle upon Tyne, NE2 4HH, UK; 3 Dept of Psychology, University of Glasgow, Glasgow, G12 8QB, UK

## 

When an individual repeatedly performs a simple sensory task, such as discrimination between similar visual stimuli, performance gradually increases until it asymptotically approaches saturation. This phenomenon is known as perceptual learning, however the neural correlates of this process are not well understood. Here we consider the results of an experiment in perceptual learning of visual contrast discrimination in cortical areas V1 and V4.

In the experiment, a Gabor (V4 recordings) or sinusoidal (V1 recordings) stimulus, with a contrast chosen at random from a set of 14 possibilities, was presented to a macaque monkey. Recordings were made using chronically implanted electrodes in a multi-unit array. The animal was tasked with determining whether the contrast was higher or lower than a control stimulus of 30% contrast, and a correct response was met with a water reward. Experimentation continued for ~20 days until performance saturated.

A population-wide linear-discriminant decoding technique based on the mean firing rates during 500ms of stimulus presentation from ~20 channels in V4 was found to achieve similar levels of performance at completing the discrimination task, and to yield a similar rate of improvement in performance, as the monkey's behavioural responses. However, the same analysis in V1 found decoder performance was the same throughout the learning process, despite the animal's improvement in performance. This suggests contrast information present in V1 remains consistent throughout learning, whilst V4 improves in its ability to readout this information from V1.
